# Polymer Hydrogel/Polybutadiene/Iron Oxide Nanoparticle Hybrid Actuators for the Characterization of NiTi Implants

**DOI:** 10.3390/ma2010207

**Published:** 2009-03-17

**Authors:** Aleksandra Jeličić, Alwin Friedrich, Katarina Jeremić, Gerd Siekmeyer, Andreas Taubert

**Affiliations:** 1 Institute of Chemistry, University of Potsdam, D-14476 Golm, Germany; 2 Faculty of Technology and Metallurgy, University of Belgrade, RS-11120 Beograd, Serbia; 3 ADMEDES Schüssler GmbH, D-75179 Pforzheim, Germany; 4 Max-Planck-Institute of Colloids and Interfaces, D-14476 Golm, Germany

**Keywords:** NiTi, nitinol, inner surface, hydrogel, polybutadiene, magnetic nanoparticles

## Abstract

One of the main issues with the use of nickel titanium alloy (NiTi) implants in cardiovascular implants (stents) is that these devices must be of very high quality in order to avoid subsequent operations due to failing stents. For small stents with diameters below ca. 2 mm, however, stent characterization is not straightforward. One of the main problems is that there are virtually no methods to characterize the interior of the NiTi tubes used for fabrication of these tiny stents. The current paper reports on a robust hybrid actuator for the characterization of NiTi tubes prior to stent fabrication. The method is based on a polymer/hydrogel/magnetic nanoparticle hybrid material and allows for the determination of the inner diameter at virtually all places in the raw NiTi tubes. Knowledge of the inner structure of the raw NiTi tubes is crucial to avoid regions that are not hollow or regions that are likely to fail due to defects inside the raw tube. The actuator enables close contact of a magnetic polymer film with the inner NiTi tube surface. The magnetic signal can be detected from outside and be used for a direct mapping of the tube interior. As a result, it is possible to detect critical regions prior to expensive and slow stent fabrication processes.

## 1. Introduction 

Nickel titanium alloys (NiTi, nitinol) are advanced materials used in medical devices. Superelasticity, shape memory, biocompatibility, MRI compatibility, and non-ferromagnetic properties are some of the characteristics that make this alloy popular in medical industry, although it is much more expensive than, for example, titanium or steel [1,2,3,4]. Arguably, the most common application of NiTi alloys are orthodontic wires and cardiovascular stents [5,6]. In cardiovascular stents, the properties of NiTi are advantageous because this material is mechanically more flexible than, for example, titanium and can respond easily to the mechanical stress exerted on the implant by active blood vessels [7]. 

NiTi cardiovascular stents, that is, fine, tubular meshes, are fabricated from NiTi tubes using laser cutting, etching, electropolishing, or knitting [5]. For the stents to function properly in the body, the starting materials (the NiTi tubes) need to (i) be hollow along the entire length of the final stent, (ii) have the same inner tube diameter throughout the whole tube to avoid pressure buildup once implanted, and (iii) be defect-free to avoid mechanical failure in the body after implantation. This implies that the starting tubes must be of good and uniform quality along the entire length of the future implant. While tubes with large diameters (down to a few millimeters) can be characterized using standard nondestructive testing (NDT) techniques, such as Eddy current testing or optical microscopy, the characterization of smaller tubes is less straightforward [8]. The majority of NDT techniques available nowadays either require too large device components to be inserted in the tubes or are limited to features larger than ca. 100 μm in size. The available techniques for the investigation of the *interior* of biomedical materials are based on radiation, such as X-ray and ultrasound, but also here, the lower resolution limit of a defect is ca. 100 μm. Moreover, these approaches require experience with data interpretation. 

Detailed knowledge of the interior of the future stent is a key parameter to determine whether or not a given piece of a tube or stent will fail or not. In particular, in small stents, the ASTM standard F04.12 (39 μm for Nitinol biomedical devices) is possibly insufficient and smaller features also need to be detected correctly [9]. This includes the detection of defects on the order of a few microns, which is smaller than what is currently technologically possible with routine characterization methods. As NiTi tubes will be used for implant manufacturing, a characterization technique should not only provide information about the interior and defects of small tubes, the technique should also be nondestructive. Otherwise, the material cannot be used for implantation. 

**Scheme 1:**
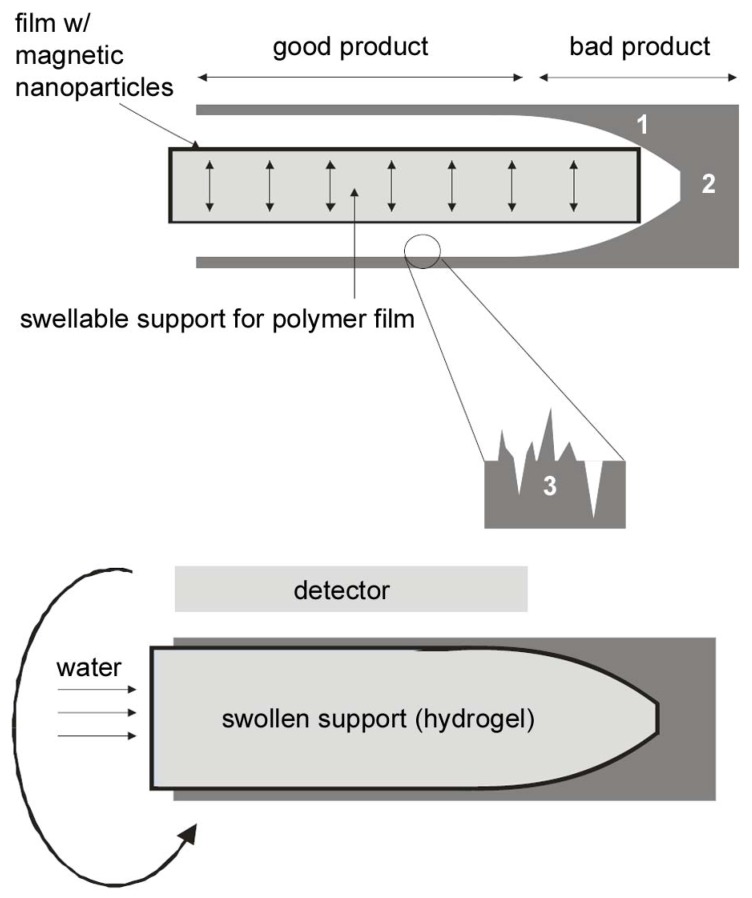
Schematic of the concept for mapping the inner surface of small NiTi tubes. (top) A dry hydrogel surrounded by an elastic polymer film containing magnetic nanoparticles (MNPs) is introduced into the tube. (bottom) The gel is swollen by the addition of an appropriate swelling agent and the MNPs embedded in the film are pushed against the inner wall. The MNPs can then be detected externally and allow for the 3D mapping of the inner surface of the tube. Bad areas with lower diameter (1), solid areas (2), and the roughness of the inner surface (3) can be detected on the “magnetic map” of the interior and the respective parts of the tube will be discarded.

The current paper describes an approach to characterize the inner surface of NiTi tubes, in particular tubes with diameters below ca. 2 mm. In short, we have prepared crosslinked, elastic polymer films containing magnetic nanoparticles (MNPs). The films can be introduced into NiTi tubes. Using commercially available superabsorbing polymers, the films are subsequently pushed against the inner tube walls via the swelling gel. As the MNPs are only located directly at the inner surface of the raw NiTi tube, the distribution of the MNPs (which can be determined via the measurement of the magnetic moment in 3D) provides a direct measure of the roughness of the inner surface, the diameter of the raw NiTi tube at any given location and the presence of defects or solid areas. As a result, bad regions or defects can be identified and the respective region of the raw tube can be discarded. Scheme 1 illustrates the concept.

## 2. Results and Discussion

### 2.1. Macroscopic film properties

Table 1 shows the composition of the films and their thickness. Table 2 summarizes their properties. Films 1 and 2, which are obtained from solutions with low PB content, contain macroscopic defects like holes and striations. These films cannot be detached from the substrate without breaking. Films 3 and 4 can be detached from the casting substrate, but film 4 is too thick for the intended use inside the NiTi tubes because defects on the order of ca. 5 μm should be detected, which can presumably not be seen anymore with a much thicker film such as film 4. As a result, further studies focused on Film 3 and variations thereof.

**Table 1:** Film composition and thickness. PB is poly(butadiene), AIBN is Azo-bis-(isobutyronitrile), TRIS is trimethylolpropane tris(3-mercaptopropionate), and Fe is iron oxide nanoparticles.

Film	[PB]/wt%	[AIBN]/wt %	[TRIS] /wt %	[Fe]/wt%	Approximate film thickness/µm
1	0.5	5.0	5.0	3.0	1.1
2	1.0	5.0	5.0	3.0	1.7
3	1.5	5.0	5.0	3.0	2.3 – 2.7
3a	1.5	-	-	-	-
3b	1.5	-	-	3.0	-
3c	1.5	5.0	5.0	-	-
4	2.0	5.0	5.0	3.0	> 4
5	1.5	5.0	5.0	1.0	-
6	1.5	5.0	5.0	3.0	2.3 – 2.7
7	1.5	5.0	5.0	6.0	-
8	1.5	5.0	1.0	3.0	2.0 – 3.0
8a	1.5	5.0	1.0	-	-
9	1.5	5.0	3.0	3.0	2.0 – 3.0
9a	1.5	5.0	3.0	-	-
10	1.5	5.0	5.0	3.0	2.3 – 2.7
10a	1.5	5.0	5.0	-	-
11	1.5	5.0	10.0	3.0	> 3.0
11a	1.5	5.0	10.0	-	-

^a^ The concentration of PB is the concentration in toluene before adding AIBN, TRIS, and MNPs. The concentration of AIBN, TRIS and Fe from MNPs is the concentration relative to the mass of polybutadiene.

Not surprisingly, Table 2 shows that uncrosslinked films cannot be removed from the substrates. This implies that the degree of crosslinking and possibly the presence of initiator (AIBN) and/or TRIS and MNPs affects the adhesion to the substrate. Kim *et al*. [10] previously investigated the influence of crosslinking degree of PB domains in mixtures of poly(styrene-butadiene-styrene) (SBS) block copolymer and tackifier on peel strength and adhesion. Both decrease with increasing crosslinking. Kim *et al*. also suggested that excess amounts of initiator act as impurities near interfaces. These impurities reduce the wettability between film and substrate. Finally, higher initiator concentration means higher radical concentration during the long mixing period (30 h) which can affect the degree of crosslinking and thus decrease the peel strength. 

**Table 2:** Solubility, macroscopic appearance, and detachability of the films listed in Table 1.

Film	Gel fraction/wt%	Macroscopic appearance	Detachable from substrate
3	56 ± 3	Mostly homogeneous Some irregularities	Yes
3a	0	Homogeneous	No, disintegrates upon removal
3b	0	Inhomogeneous	No, disintegrates upon removal
3c	< 20	Mostly homogeneous Some irregularities	Only after 3 weeks of exposure to daylight
5	37 ± 7	Mostly homogeneous Some irregularities	Partly more fragile than films 6 and 7
6	56 ± 3	Mostly homogeneous Some irregularities	Yes
7	58 ± 3	Mostly homogeneous Some irregularities	Yes
8	< 20	Inhomogeneous	No
8a	< 20	Inhomogeneous	No
9	< 50	Inhomogeneous	No
9a	< 20	Inhomogeneous	No
10	56 ± 3	Mostly homogeneous Some irregularities	Yes
10a	< 20	Mostly homogeneous Some irregularities	Only after 3 weeks of exposure to daylight
11	73 ± 5	Mostly homogeneous Some irregularities	Yes
11a	< 20	Mostly homogeneous Some irregularities	Only after 3 weeks of exposure to daylight

Table 2 also shows that film 3c slowly crosslinks upon exposure to light and that the presence of iron oxide nanoparticles seems to enhance the crosslinking process. Indeed, El-Tantawy *et al*. [11] reported that Fe_3_O_4_ promotes crosslinking and that the degree of crosslinking increases as the concentration of Fe_3_O_4_ increases. In the current case, the presence of iron oxide nanoparticles is therefore advantageous for two reasons. First, it enhances the crosslinking reaction and second, it furnishes the films with a magnetic property, which is desired for the final application in the NiTi tubes, Scheme 1. 

For this latter point, a strong magnetic signal and hence a high concentration of the magnetic nanoparticles is desirable. Besides, the films need to be homogeneous and stable under regular handling conditions. To determine the influence of MNP concentration on film properties, films with different MNP concentrations were prepared. As film 3 has the most appropriate properties among films of different thickness, a PB concentration of 1.5 wt % in toluene was chosen for preparation of films 5 to 7, Table 1 and Table 2.

Table 2 shows that the gel fraction increases as MNP concentration increases. However, although film 7 contains twice as many iron oxide particles as film 6, the gel fraction does not increase over ca. 55 to 58 %, suggesting that there is a maximum degree of crosslinking which cannot be exceeded under the current conditions. Besides the high degree of crosslinking and high loading of magnetic nanoparticles, the homogeneity and stability of the films is important. Films 5 to 7 can be detached from the substrate, but film 5 is less stable and only partly detaches. 

The effect of TRIS concentration was probed with films 8 to 11. The PB and Fe (from MNPs) concentrations are constant at 1.5 wt% and 3 wt%, because films 3 and 6 have been found to exhibit a suitable film thickness and homogeneous MNP distribution. Films 8 and 9 have an irregular macroscopic appearance, while films 10 and 11 are macroscopically homogeneous. If the MNPs are omitted during film formation (films 8a to 11a), but the TRIS concentrations are kept constant, the films only poorly crosslink and cannot be removed from the substrate. Only after three weeks of daylight irradiation, the degree of crosslinking (via an uncontrolled crosslinking process initiated by various irradiation conditions) is high enough to form detachable films. This observation illustrates that the MNPs not only provide the magnetic moment needed for the device to finally function, but also enhance the crosslinking reaction, similar to El Tantawy *et al*. [11] Overall, the presence of the irregularities described above (striations, holes, etc.) seems to depend on the TRIS concentration rather than the MNPs. Low TRIS concentrations lead to films of poor quality, whereas more strongly crosslinked films with higher TRIS concentrations are more homogeneous.

**Figure 1:**
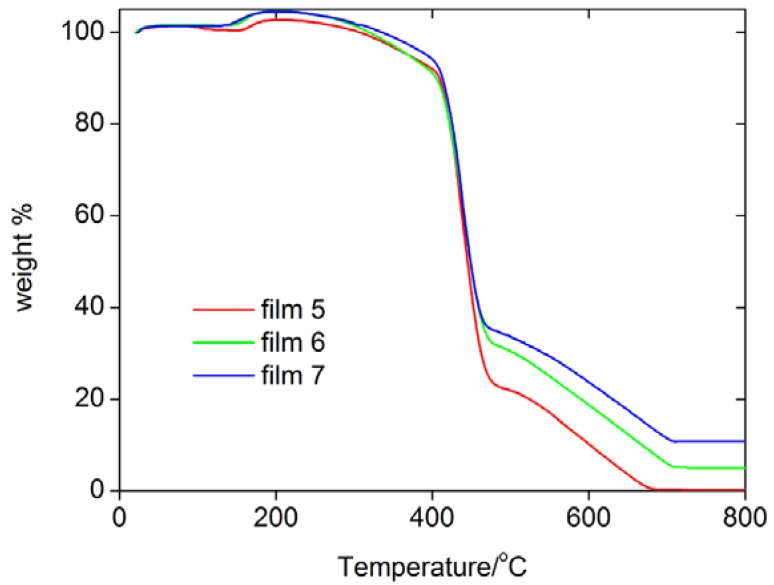
TGA curves of some films.

Figure 1 shows thermogravimetric analysis (TGA) data of some films. TGA shows that they essentially behave the same, that is, the incorporation of the MNPs does not significantly alter the thermal stability of the resulting hybrid films. TGA finds one rapid weight loss between 420 and 450 ºC, which is followed by a slower weight loss between 500 and 700 ºC. Differential thermal analysis (DTA) shows that both processes are exothermic processes, which suggests that the polymer is thermally decomposing and the remains are burning at higher temperatures. The remaining mass at 800 ºC differs depending on the weight of iron oxide particles loaded in the films. 

### 2.2. Microscopic film homogeneity and MNP distribution

Essentially, films 3, 5, 6, 7, 10, and 11 have favorable macroscopic properties for the intended application: homogeneous morphology, high gel fraction, and easy detachability from the substrate. However, to determine the inner structure of the NiTi tubes, the distribution of the MNPs must also be homogeneous on a microscopic level and the films must also be microscopically homogeneous and stable. 

**Figure 2:**
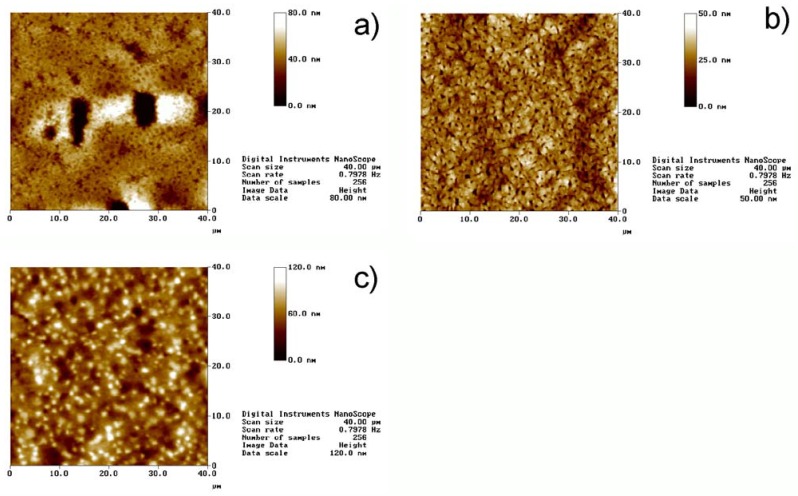
AFM images of different films. a) Film 5, b) film 6, and c) film 7. Note that the height scales are different. The defect shown in Figure 2a is atypical for Film 5 and was only observed very rarely. Regular surfaces of film 5 look like the area in the top third of the image. The defect was chosen to show the dimensions of such defects.

Figure 2 shows representative atomic force microscopy (AFM) images of films with different MNP fractions. Macroscopically inhomogeneous films show microscopic inhomogeneities like dents and elevated areas. The diameters of these features are up to ca. 20 μm in diameter and the depth or height reaches up to 500 nm. In contrast, macroscopically homogeneous films exhibit uniform surface topographies with only few smaller dents with a maximum depth of ca. 300 nm. The mean square (Rms) roughness for all the films is between 5 and 80 nm. The defect shown in Figure 1a is an atypical case, as usually film 5 and also film 6 do not exhibit large defects. However, the image illustrates well the types of defects that can occur.

Film 7, that is, the film with the highest MNP concentration, exhibits numerous “dots” in the AFM images. These are assigned to some clustering of the MNPs at or near the surface, which is consistent with scanning electron microscopy data of the same films (data not shown). Indeed, Wilson *et al.* [12] have investigated the behavior of iron oxide particles in nanocomposites obtained by melt-blending and confirmed that nanoparticles tend to migrate under the surface. Gass *et al*. [13] confirmed the same behavior in spin-coated composites. We thus conclude that particle migration occurs in our films as well, including in film 7 where the concentration of MNPs is so high that numerous clustered particles form even on the surface. 

**Figure 3:**
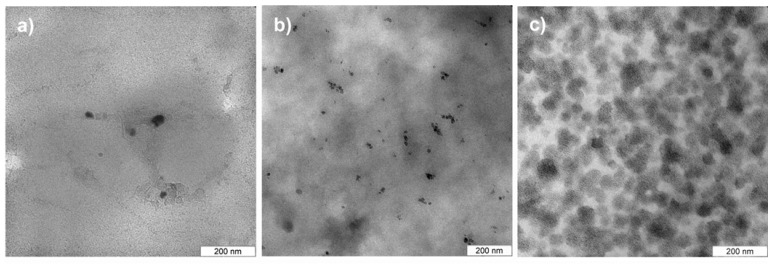
TEM images of MNP distribution in a) film 5, b) film 6, and c) film 7. Sections are longitudinal thin sections, that is, sections in the plane of the film.

Figure 3 shows representative transmission electron microscopy (TEM) images of longitudinal thin sections of the films. TEM confirms AFM and shows that there are only a few nanoparticles in film 5. Particle aggregates consist of no more than 10 particles and are below 200 nm in size. Occasionally there are also larger areas without nanoparticles. Film 6 contains nanoparticles in all areas of the films studied. In film 7, the high nanoparticle concentration results in numerous agglomerates of around 80 nm in diameter. The agglomerates are connected, but there are other areas with very low nanoparticle density. This suggests that the AFM images indeed show MNP clusters at or near the surface of film 7. Overall, AFM and TEM show that film 6 has the best properties, that is, uniform MNP distribution, smooth and uniform micromorphology and uniform macromorphology for the desired application. 

### 2.3. Swelling of the gels

Hydrogels are used to provide inside pressure in the tube and push the polymer/MNP film to the tube walls. Several requirements need to be met: (i) the polymers need to swell fast to avoid time-consuming characterization procedures, (ii) the polymers need to swell uniformly to provide a uniform distribution of the MNPs on the inner tube walls, (iii) the polymers need to swell sufficiently to provide strong contact between the tube walls and the polymer film. 

**Figure 4:**
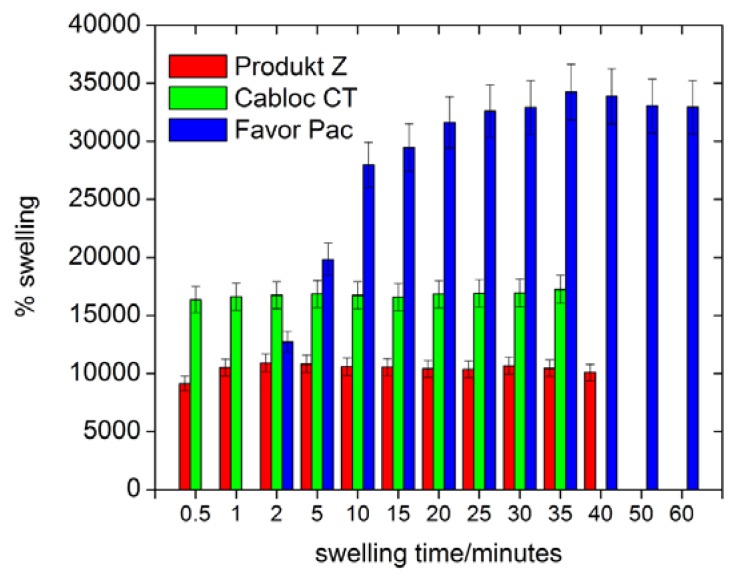
Swelling of the polymers in deionized water vs. time.

**Figure 5:**
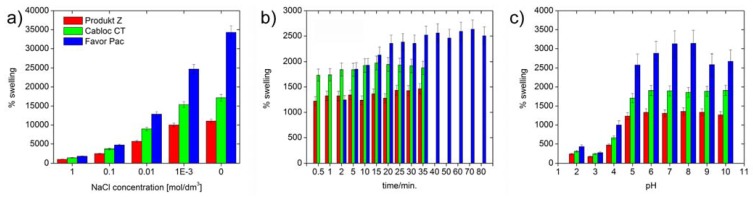
Swelling of the three polymers in deionized water as a function of time: a) maximum degree of swelling vs. ionic strength, b) swelling rate for 0.5 M NaCl. Compare Figure 4 for swelling in pure water, c) maximum swelling at different pH values.

Figure 4 shows that all polymers swell by at least 8000 % in deionized water. Figure 5 also shows that Produkt Z and Cabloc CT swell rather quickly and within a few minutes reach their final degrees of swelling. Favor pac 230 swells more, but requires ca. 30 minutes to reach the final degree of swelling. 

Figure 5 shows that, not surprisingly, the swelling is strongly reduced with ionic strength, but the *rates* of swelling are not affected by the ionic strength. As all polymers are crosslinked polyacids, their degrees of swelling at pH 5 and higher are roughly unchanged, but the hydrogels collapse at pH 4 and lower and a much weaker swelling is observed. 

**Figure 6:**
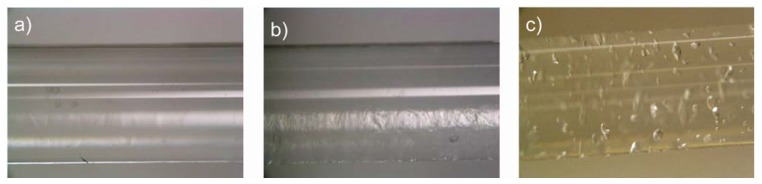
Representative photographs of hydrogels swollen in 2 mm diameter glass capillaries with deionized water. (a) Cabloc CT, (b) Produkt Z 1069, and (d) Favor pac 230. White lines on the tubes are reflections on the glass from the lamp used to illuminate the samples.

For the final device to function properly, complete filling of the tubes and a clean contact with the inner surface of the tubes is required. Bubbles and other defects are to be avoided. Figure 6 shows photographs of the three polymers in glass tubes with the same diameters as the final implants (2 mm). Cabloc CT completely fills the inner part of the capillary. Produkt Z also fills the capillary, but some contrast indicative of a residual roughness is observed here. Favor pac remains partially unswollen and has numerous cavities. As a result, Cabloc CT is most suited for the actuator and we have therefore focused on Cabloc CT for the remainder of the study.

### 2.4. Implementation of the device 

As discussed above, film 10 (which has the same composition and properties as films 3 and 6) shows the best characteristics in terms of mechanical properties, homogeneity, and MNP loading. From the polymer hydrogels, Cabloc CT shows best filling characteristics. These two components were therefore chosen for a prototype of the final device. 

**Figure 7:**
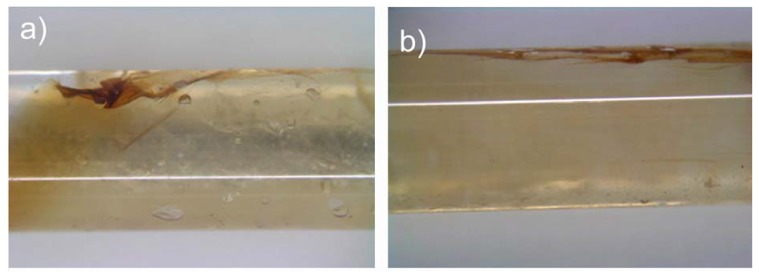
Photographs of the hybrid actuator assembled from films and swollen Cabloc CT hydrogels in a 2 mm diameter glass tube. There are two common defects: (a) the film adheres well to the inner surface but some parts of the film overlap. (b) At lower loading of hydrogel, there are bubbles between the wall and the film. Both structures will result in wrong reconstructions of the inner surface. White lines on the tubes are reflection on the glass from the lamp used to illuminate the samples.

Figure 7 shows typical results, including the two most common defects. The film (brownish) inserted into the glass capillaries adheres well to the capillary walls. However, overlapping of the film can be also noticed, as well as some cavities between the wall and film due to lack of hydrogel at that particular place. As a result, Figure 7 shows that the magnetic film can be introduced and pushed against the tube wall by the Cabloc CT filling. Figure 7, however, also shows that the implementation is not perfect yet. There is a need for further optimization, in particular with respect to the film-tube contact and folding of the film. 

In summary, the current study shows that our actuator is interesting for the analysis of tubular structures, but also that there is need for improvement. Approaches for improvement are (i) fabrication of more homogeneous films by using more controlled conditions such as spin coating or solution casting under better evaporation control, (ii) optimization of the MNP dispersion prior and during film formation, (iii) fabrication of preformed hydrogel components in a mold instead of using superabsorbent polymer powders, and (iv), on the longer run, a synthetic approach leading to a polymer hydrogel “monolith” where the magnetic film can be covalently attached to the surface as smooth film. Especially the last approach will help avoiding the effects observed in Figure 7: if the MNP/polymer film is attached to the central hydrogel part (which has been molded into a certain shape), folding will be prevented. Finally, the swelling of the central hydrogel must be controlled more accurately in order to avoid bubbles. 

## 3. Experimental Section

### 3.1. Materials

Polybutadiene (PB, Mw ca. 420,000 g/mol; 36% *cis*-1,4; 55% *trans*-1,4; 9% vinyl), magnetic iron oxide nanoparticles stabilized with oleic acid and dispersed in heptane (0.5 to 0.7% as Fe, 6.5 ± 3.0 nm Fe-Oxide-Nucleus, 10-40 nm hydrodynamic diameter), trimethylolpropane tris(3-mercaptopropionate) (TRIS), and 2,2'-azo-bis-isobutyronitrile (AIBN) were obtained from Aldrich and used without further purification. Cover slips (20 x 20 mm, thickness 0.13 - 0.16 mm) were obtained from Menzel-Gläser/VWR. Superabsorbing polymers (Favor pac 230, Cabloc CT, Produkt Z 1069) were supplied by Degussa. The polymers are partially neutralized, grafted copolymers of crosslinked polyvinyl alcohol and polyacrylic acid. Favor pac 230 has a particle size of 150 to 850 µm, and dry water content of < 2 %. Cabloc CT has a particle size of 100 to 300 µm and dry water content of 5 ± 2 %. Produkt Z 1069 has a particle size of 50 to 200 µm and a dry water content of 5 ± 2 %.

### 3.2. Film preparation

Polymer films were prepared by solution casting from toluene onto microscopy cover slips. In a typical experiment, a polybutadiene (PB) solution in toluene (10 mL) was mixed with solutions of AIBN and TRIS in toluene. Then a dispersion of MNPs in heptane was added and the mixture agitated in a shaker (stirring with a magnetic stirrer causes the MNPs to stick to the magnetic bar) for ca. 30 h in the dark. 200 µL of the dispersion were deposited on cover slips placed in Petri dishes covered with aluminum foil. Evaporation was roughly adjusted by the numbers of holes in the aluminum foil or by placing the samples directly in a vacuum oven. All experiments were done at room temperature. After solvent evaporation, the films were dried overnight in a vacuum oven at room temperature. See Table 1 and Table 2 for film details. The glass substrates were studied via atomic force microscopy and optical microscopy after detaching of the films. There were no signs of residuals in the crosslinked films, that is, in the films, where Table 2 states “detachable from substrate”. The non-crosslinked films did show quite significant material remaining on the glass.

### 3.3. Microscopy

Atomic force microscopy (AFM) was done at ambient conditions on a Veeco NanoScope IIIa in tapping mode with silicon nitride tips (Nano World, T = 4.6 µm, L = 160 µm, Force constant = 42 N/m, Resonance frequency = 285 kHz). Film thickness was determined by scratching the sample with a needle and subsequent measurements of the vertical distance between the glass substrate and film surface. Scanning electron microscopy (SEM) was done on films sputter-coated with gold using a Philips XL-30 ESEM operated at 3 kV. Transmission electron microscopy (TEM) of thin sections of the polymer films was done on a Zeiss EM 912 Omega microscope at 120 kV. For microtomy, samples were embedded in LR White in Gelatin Capsules and polymerized at 60 ºC. 70 to 100 nm sections were made on a Leica Ultracut UCT with a diamond knife and collected on carbon-coated copper grids. 

### 3.4. Thermogravimetric Analysis (TGA) and Differential Thermal Analysis (DTA)

TGA and DTA measurements were done in a stationary air atmosphere (no purge) from 30 to 800 ºC using a Linseis L81 thermal analyzer (Linseis, Germany) working in vertical mode. The heating rate was 10 ºC/min. and Al_2_O_3_ was used as reference.

### 3.5. Determination of the gel fraction 

Immediately after casting and drying to constant mass, the films were dissolved in toluene for 24 h at room temperature. The insoluble fraction was recovered by filtration and dried *in vacuo* at room temperature to constant weight. The gel fraction was determined via φ_gel_ = M_di_/M_ini_, where M_di_ is the mass of the dry, insoluble fraction and M_ini_ is the initial mass of the starting material. 

### 3.6. Degree of swelling

The swelling ratio R = [(W_s_ – W_d_)/W_d_] × 100, where W_s_ and W_d_ are weights of the swollen and dry gel, respectively, was determined for Favor pac 230, Cabloc CT, Produkt Z 1069 as a function of swelling time, ionic strength, and pH. For determination of the effect of time and ionic strength, 100 mg of hydrogel were left to swell in 100 mL of water or aqueous NaCl with different ionic strengths for various amounts of time. Hydrogels were filtered off and residual water was picked up with filter paper and the hydrogel was weighed. For measuring the swelling dependence of pH, 100 mg of polymer were left to swell in 100 mL of 0.5 M NaCl for 24 h and then the pH was adjusted by adding NaOH and HCl. 0.5 M NaCl was necessary to suppress the influence of ionic strength on swelling when adding HCl or NaOH. 

### 3.7. Setup implementation

Proof of concept experiments for the actuator were made with a glass tube model of 2 mm diameter. Dry hydrogel was deposited on a syringe needle, the PB/MNP film was wrapped around the dry gel, and the whole construct was inserted into glass capillaries with an outer diameter of 2 mm and a length of 20 mm. Then water was injected and the gel was left to swell for approximately 20 minutes. 

## 4. Conclusions

The current paper shows that composite actuators of crosslinked polybutadiene/MNP thin films and polymer hydrogels can be used for the characterization of the inner structure of (model) implant tubes. The best performance is found for polymer films cast from a 1.5 wt % PB solution, an AIBN concentration of 5.0 wt%, a TRIS concentration of 5.0 wt%, and an MNP concentration of 3.0 wt%. Higher concentrations of MNPs, although desirable for improved performance, lead to agglomerates and rougher films. Cabloc CT is the preferred hydrogel component. Overall the current paper shows that the approach is a viable pathway for the characterization of the interior of small tubes. However, the film preparation, the insertion into the tube, and the shape control of the hydrogel component will have to be optimized for reproducible performance.
